# Improvement of speech perception following articulatory-target based production training for second language acquisition

**DOI:** 10.3389/fnhum.2026.1743034

**Published:** 2026-04-13

**Authors:** Atsuo Suemitsu, Takayuki Ito, Jianwu Dang, Yukiko Nota, Kikuo Maekawa, Mark Tiede

**Affiliations:** 1 Department of Information Science and Technology, Faculty of Information Science and Technology, Hokkaido University of Science, Sapporo, Japan; 2 Univ. Grenoble Alpes, CNRS, Grenoble INP, GIPSA-lab, Grenoble, France; 3 Shenzhen Institute of Advanced Technology, Chinese Academy of Sciences (CAS), Shenzhen, China; 4 Advanced Language Science (E3P) Research Center, National Institute for Japanese Language and Linguistics, Tachikawa, Japan; 5 National Institute for Japanese Language and Linguistics, Tachikawa, Japan; 6 Department of Psychiatry, Yale University, New Haven, CT, United States

**Keywords:** articulation training, electromagnetic articulography, EMA, Pronunciation, real-time visual feedback system, L2 learning

## Abstract

Direct training of articulatory movements can be an effective and efficient tool for second language learning. We have previously developed an articulatory target-based training method using electromagnetic articulography for second language pronunciation. In this training, an estimate of a participant's target midsagittal tongue posture is presented on a display together with the real-time position of their tongue, and used as feedback for adjusting their tongue position to the target position. We showed that Japanese participants improved their pronunciation of American English vowel /æ/, which is not in the Japanese vowel inventory, toward more native-like pronunciation following the training. The current study investigates whether this articulatory target-based training approach, which demonstrably improves production, enhances perception of the corresponding speech sounds. Seven Japanese male speakers participated, performing an ABX perceptual test before and after training, contrasting minimal pairs: /æ/-/ɑ/, /æ/-/ʌ/, and /ɑ/-/ʌ/ within consonant-vowel-consonant words. Results showed that, in spite of the large range of observed variability in individual participants' responses to the articulatory target-based training, overall, there were significant improvements in both production and perception of /æ/. Our findings suggest that motor processing related to speech production contributes to the improvement of speech perception for the second language acquisition, and shows that articulatory target-based training approaches can be useful for enhancing both production and perception in second language acquisition.

## 1 Introduction

Acquiring a second language (L2), is challenging for adult L2 learners, especially mastering accurate pronunciation and perception. Even when high levels of proficiency in grammar and vocabulary are achieved, reaching native-like pronunciation and perception frequently remains elusive. This difficulty is often attributed to the influence of the L2 learner's native language (L1), a phenomenon known as L1 interference (Gass and Selinker, 2008). Phonological categories formed early in life act like a perceptual filter for L2 sounds (Kwon and Starr, 2023). In speech perception, L2 learners perceive unfamiliar L2 phones as their closest L1 categories, which, as explained by the Perceptual Assimilation Model (Best, 1995), function as “perceptual magnets” (Kuhl, 1991). When an L2 contrast is absent from the L1 inventory, discrimination is difficult since novel sounds are assimilated to existing L1 categories (Best and Tyler, 2007). This perceptual limitation typically carries over to production: contrasts that are not perceived reliably are rarely produced accurately. Some novel sounds may be reliably perceived due to large phonological distance from the native inventory, yet those may be still difficult to produce because of the unfamiliar articulatory gestures needed to produce them (Bohn and Flege, 1997; Derwing and Munro, 2015).

Traditional pronunciation teaching has emphasized listening and imitation. While this strategy can be helpful, its effectiveness is limited when L1 phonotactics interfere with L2 learners perceiving the target contrast. If L2 learners cannot perceive the relevant difference, imitation becomes trial-and-error without a clear route to correction. Providing descriptive articulatory information can be beneficial, but depends on L2 learners being able to interpret that information in order to successfully adjust their articulation. Consequently, the effectiveness of this production training can vary considerably depending on individual ability to modify their own articulatory movement as intended based on descriptive articulatory information. Articulatory-based visual feedback, using technologies like ultrasound and Electromagnetic Articulography (EMA), can overcome this issue by providing real-time information about L2 learners' own speech gestures (Bliss et al., 2018). Ultrasound imaging, for instance, has proven effective in helping L2 learners by enabling them to align their articulatory movements with target descriptive information, ultimately facilitating the acquisition of the French /y/-/u/ contrast for Japanese L2 learners (Pillot-Loiseau et al., 2013) and in various English sounds (Gick et al., 2008). EMA systems, which track sensors placed on the tongue and other articulators, have also been useful in L2 production training paradigms by presenting target articulatory information as visual guidance (Levitt and Katz, 2010; Suemitsu et al., 2015). This approach allows L2 learners to adjust their articulatory positions directly from visual guidance without the need for any descriptive articulatory information. Since providing visual guidance for pronunciation adjustment offers an explicit and intuitive pathway (Badin et al., 2010), these methods, together with other 3D visual feedback systems (Katz et al., 2014), can be a useful tool for L2 production training and help mitigate L1 interference.

Explicit production training that focuses on underlying articulatory mechanics can also improve perceptual acuity, due to the coupling between speech perception and production. The Motor Theory of Speech Perception proposes that speech is perceived with reference to the gestures used to produce it (Liberman et al., 1967). Although the original formulation remains controversial, substantial evidence now supports a tight sensorimotor linkage. In cortical processing, the premotor areas corresponding to speech production is also involved in speech perception (Wilson et al., 2004; D'Ausilio et al., 2009; Möttönen and Watkins, 2009). At the behavioral level, articulatory movement during speech production has been shown to modify speech perception when the articulatory movement is executed in conjunction with the presentation of speech sounds (Sams et al., 2005; Mochida et al., 2013). The relationship is bidirectional and dynamic: weakness in one domain is frequently mirrored in the other, whereas improvement in one can facilitate gains in the other. For example, auditory acuity in speech perception has been shown to be positively correlated with the related orofacial somatosensory acuity (Perkell et al., 2004; Ghosh et al., 2010). Individuals showing less within-phoneme variability and greater between-phoneme distances in production demonstrated better auditory discrimination performance (Franken et al., 2017). The vowel categorization boundary in speech perception is positively correlated with the corresponding boundary in speech production, which is determined based on the variability of the vowel production (Chao et al., 2019). Recent research shows that manipulating one system can also calibrate or adjust the other. Altered auditory feedback studies, where speakers hear a real-time, perturbed version of their own voice, demonstrate that adaptation in production can recalibrate speech perception (Shiller et al., 2009; Lametti et al., 2010; Ohashi and Ito, 2019). These findings suggest that motor adaptation and learning contribute to maintaining and tuning perceptual categories. Based on this framework, we hypothesize that explicit motor learning involved in achieving the correct articulatory posture can not only improve production accuracy but also refine the perceptual boundaries of this newly learned sound category.

Direct training of gestures, through the target-matching visual feedback approach mentioned above, may be potentially useful by providing the most salient information for establishing a new perceptual category. Although laboratory-level speech adaptation procedures provide evidence of perceptual modulation following motor adaptation, findings have been contradictory in L2 training situations. While some studies report perceptual improvements following production training (Kartushina et al., 2015; Linebaugh and Roche, 2015; Li, 2024; Stratton, 2025), others have reported no improvement (Wong, 2013) or even detrimental effects (Baese-Berk and Samuel, 2016). This contradiction is also found with the ultrasound imaging approach, with some studies showing improvement (Chang, 2023) and others no effect (Tateishi and Winters, 2013). Given these inconsistent findings, it remains crucial to clarify under what specific training conditions or approaches perceptual improvements can be effectively induced.

In our previous work, we proposed and developed an EMA-based real-time visual feedback system for L2 pronunciation training (Suemitsu et al., 2015). This procedure relies on EMA, which provides high spatial and temporal resolution of the articulator positions sampled by its sensors. This facilitates the estimation of speaker-specific articulatory positions associated with novel speech targets, and the presentation of these targets together with a real-time display of the user's current articulatory position and the palate. As a result, participants can visually monitor adjustments to their articulators while attempting to match the target positions, which is difficult to achieve using other articulatory measurement devices. Ultrasound, for example, shows only tongue position relative to the probe (which displaces with jaw movement), rather than showing the actual tongue position within the context of the palatal hard structure. Although EMA sensors provide only a sparse representation of anterior tongue position (i.e., three or four sampled points), they also track jaw and lips, and are readily amenable to real-time correction for head movement, making it visually less confusing for participants to match the novel prototype positions. Estimation of speaker-specific targets is also more straightforward when only a limited number of articulatory reference locations need to be considered. We applied this system to Japanese learners of American English (AE) to train the front low vowel /æ/, which does not exist in the Japanese vowel system. Japanese speakers often assimilate /æ/ to /a/, resulting in productions that can be ambiguous for native English listeners. We have demonstrated that this system improved Japanese participants' productions toward more native-like sounds within a relatively short training period. This improvement was observed in training sessions with both articulatory and acoustic cues presented, and with articulatory cues alone, but not training solely with acoustic cues (model sounds produced by AE speakers). This suggests that direct adjustment to the articulatory target is an effective and efficient tool for L2 production learning, rather than traditional training solely with acoustic cues. Although studies investigating perceptual improvement from L2 production training have shown mixed results, we expect the target-based direct training approach we describe to be effective for modulating perception of the trained L2 sounds.

The previous studies have often applied a combination of articulatory guidance with traditional auditory exemplars (e.g., Kartushina et al., 2015; Chang, 2023). The inclusion of an auditory component makes it difficult to isolate the specific motor contribution to perceptual change. Since our EMA-based approach demonstrated that articulatory target-based (ATB) training alone without auditory exemplars improved production performance to a similar extent as training with auditory exemplars, employing ATB training alone allows us to directly examine whether perceptual improvement can arise from motor-based rather than acoustic- based learning.

The current study investigates the effects of this ATB production training on speech perception. We examined whether the training, in the complete absence of native auditory exemplars, could improve participants' perceptual discrimination ability. To this end, we employed a pre-/post-design in which monolingual Japanese speakers participated in a short ATB training session for the AE vowel /æ/. Their ability to produce the vowel and, critically, their ability to perceptually discriminate it from neighboring vowels (/ɑ/ and /ʌ/) were assessed before and after the training. By isolating the training to the articulatory-motor domain, we aim to offer a clearer test of the hypothesis that motor processes related to speech production directly contribute to the refinement of speech perception in L2 acquisition. For this purpose, we employed training solely with motor cues in the absence of any acoustic cues. Together with the results of our previous study, the results of this study provide evidence that motor-based training techniques can be an effective and efficient tool for improving both productive and receptive phonological skills in L2 learners.

## 2 Methods

### 2.1 Participants

Seven male Japanese monolingual speakers participated in the study. Participants ranged in age from 18 to 30 years old, with no reported hearing or speech disorders. None had lived abroad or had extensive exposure to native English-speaking environments. The experiment was carried out at two sites: Japan Advanced Institute of Science and Technology (JAIST) and Sapporo University of Health Sciences. Three participants (M1, M2, and M3) were tested at the former site and the rest (M4, M5, M6, and M7), at the latter site. All participants provided informed consent, and the study was approved by the ethical review board in each institute (JAIST approval number: 28–007, approval number of Sapporo University of Health Sciences: 016005).

### 2.2 EMA-based, real-time, visual feedback system for articulatory target-based training

**Figure 1:**
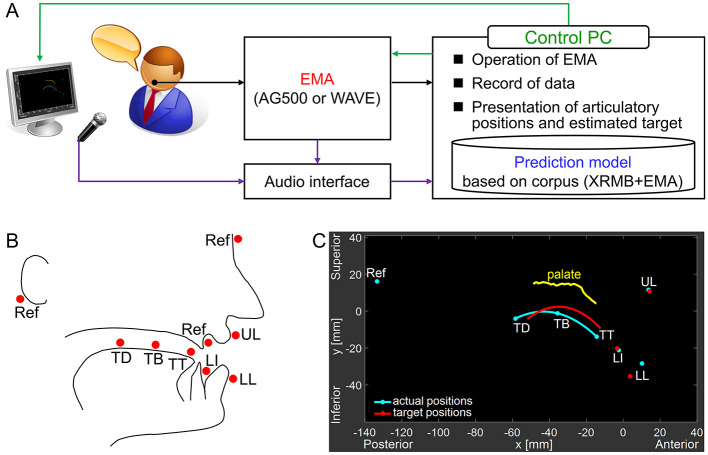
Overview of the EMA-based real-time articulatory training system. **(A)** Schematic diagram of the system. **(B)** EMA sensor placement: tongue tip (TT), tongue blade (TB), tongue dorsum (TD), lower incisors (LI), upper lip (UL), lower lip (LL), with reference sensors (Ref) attached to the nasion, upper incisors, and mastoid processes. **(C)** Example of the real-time visual feedback display, showing the tongue contour interpolated from the three tongue sensors (cyan line), the pre-recorded palate trace (yellow line), and the participant-specific predicted target positions for the AE vowel /æ/ (red line and points). Labels in the figure and in the axes are only for the illustration purpose, and these were not shown on the actual experiment.

Figure 1A shows a schematic diagram of the system using an EMA to present articulatory positions in real time, along with speaker-specific target positions estimated from speaker acoustics and articulatory data. Both our originally developed system (Suemitsu et al., 2015) and current sessions conducted at JAIST use the AG500 (Carstens Medizinelektronik). For sessions at Sapporo University of Health Sciences, a WAVE (Northern Digital Inc.) system is used. The visual feedback module, developed in MATLAB (MathWorks), is the same in both configurations.

In the current study, we applied this system for pronunciation training of the AE vowel /æ/, lacking among Japanese vowels, to Japanese learners of AE. The visual feedback and target estimation parts of this system are described in the following paragraphs.

EMA sensors were placed on the tongue tip (TT), blade (TB), and dorsum (TD), lower incisors (LI), upper lip (UL), and lower lip (LL), together with reference sensors on the upper incisors, nasion, and mastoid processes, as shown in Figure 1B. All sensors, except for the mastoid references, were on the speaker's midsagittal plane. The reference sensors were used to correct for head movement and align data to a speaker-centric coordinate system based on their occlusal plane. Synchronized speech audio was co-recorded using a directional microphone (RODE NTG-3).

Articulatory movement and speech acoustics were digitized at sampling rates of 200 Hz and 16 kHz for the AG500 case, and 100 Hz and 22.05 kHz in the WAVE case. These differences in recording parameters did not affect the quality of recorded data since the Nyquist rates are higher than the frequency ranges of interest: articulatory movements at normal speaking rates are typically less than 6 Hz, and acoustical power of vowel production is primarily concentrated below 8 kHz. In addition, these differences also had no effect on real-time visual presentation since the sampling frequencies of articulatory movement are higher than the refresh rate of visual presentation (20 Hz, as described below).

Figure 1C illustrates an example of the real-time visual feedback display. For visualization, the sensor position data were transformed into a participant-specific coordinate system, corrected for head movement and aligned to their occlusal plane. This transformation was calculated every 50 ms, limiting the visual feedback update rate to 20 Hz. The delay between actual and visualized movement was imperceptible to the participants. The tongue surface contour was generated using cubic spline interpolation through the three tongue sensor positions (cyan line in the figure). The palate shape (yellow line) was recorded during the initial Preparation phase (Section 2.3.1.1 below), and displayed during training to provide a fixed reference for articulatory position.

To specifically tailor production targets to each participant's individual vocal tract morphology, speaker-specific target articulatory positions for /æ/ were estimated by employing a multiple linear regression model trained on data from native speakers of AE. The prediction model was constructed using acoustic and kinematic data from 40 native speakers of AE (19 males and 21 females) drawn from the University of Wisconsin X-ray microbeam (XRMB) speech production corpus (Westbury, 1994). This core dataset was augmented by the inclusion of EMA data from an additional 9 native speakers (5 males and 4 females) collected at Haskins Laboratories. We included data from both genders to obtain an inclusive prediction model for both genders. (While known morphological differences across gender (e.g., vocal tract length, proportionally longer pharynx) suggest that gender-specific models could potentially provide greater accuracy, our available training data was not sufficient to provide stable convergent models distinct by gender.) The model utilized the AE vowels /ɑ/, /i/, and /u/ in each AE speaker as predictor variables. These vowels were chosen due to their reasonable acoustic and articulatory overlap with Japanese vowels /a/, /i/, and /ɯ/, a crucial consideration for understanding cross-linguistic influences on vowel production. Importantly, the acoustic characteristics of the Japanese participants' vowel productions overlapped with those of AE speakers used in this study, supporting the validity of this cross-linguistic mapping (see Supplementary Figure 1).

Twelve distinct multiple regression models were generated for the prediction model, corresponding to the respective posterior/anterior (X) and inferior/superior (Y) coordinates of six articulatory attachment points (TT, TB, TD, LI, UL, and LL) (EMA lateral and angular information were not used). These models were built using a stepwise selection procedure applied to a set of predictor variables. These predictors included: the first (F1) and second (F2) formant frequencies, X and Y coordinate values of the six articulatory attachment points (TT, TB, TD, LI, UL, and LL) for the AE vowels /ɑ/, /i/, and /u/, the area of the triangle formed by these three vowels in the F1 × F2 acoustic space, and the area of the triangle defined by the TDxy positions associated with these same vowels. In total, 44 predictors were applied. Because the primary objective of this prediction model was to obtain better predictive performance rather than to characterize contributions of specific variables, we chose this stepwise selection procedure because it resulted higher estimation accuracy than alternative (forced-entry) approaches. Note that the TB position was estimated by calculating the midpoint between the T2 (mid-ventral) and T3 (mid-dorsal) pellets from the Wisconsin XRMB corpus. The TT and TD positions corresponded directly to the T1 (ventral) and T4 (dorsal) pellets, respectively. For each participant, these regression models were applied to the prerecorded baseline productions of the Japanese vowels /a/, /i/, and /ɯ/ to estimate their individual articulatory target positions for AE vowel /æ/.

### 2.3 Experimental procedure

**Figure 2:**
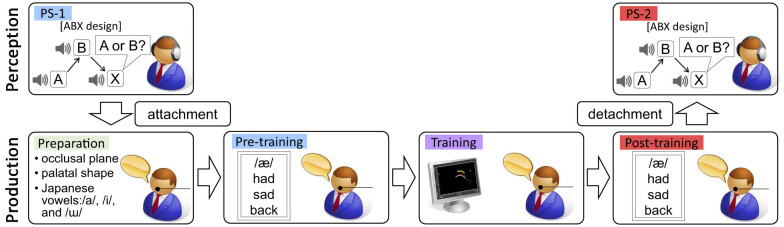
Schematic overview of the experimental procedure. The production session consisted of four phases: preparation (sensor setup, calibration, and baseline vowel recordings), pre-training (baseline production of target stimuli including the vowel /æ/), training (ATB training using real-time EMA feedback without exposure to any native auditory models), and post-training (production of the same stimuli without target display). The perception session employed an ABX discrimination paradigm with minimal vowel pairs (/æ/–/ɑ/, /æ/–/ʌ/, and /ɑ/–/ʌ/), conducted both before and after the production session.

To examine the effect of the production training on speech perception, we carried out separate production and perception sessions in the following sequence: 1) perception session (PS-1); 2) production session; and 3) perception session (PS-2), see Figure 2 for details. The production session used the ATB feedback approach established in our previous study (Suemitsu et al., 2015). Articulatory cues were provided based on the prediction model described above, and acoustic cues were model sounds produced by AE native speakers. To eliminate the possibility that acoustic cues presented during training may affect changes in speech perception, here we applied the training solely with articulatory cues, in which the participants were exposed only to their own produced sounds, but not to the model vowel sounds produced by AE speakers. The articulatory training phase lasted 10–15 minutes. The perception session assessed participants' perceptual discrimination of the target vowel using an ABX vowel discrimination task. Note that participants did not wear any EMA sensors used in the production session during either of the perception sessions. Following these sessions, a brief follow-up interview was conducted to collect participants' impressions. The same testing environment was used for both speech perception and production to minimize potential confounding variables related to room acoustics or external distractions.

#### 2.3.1 Production session

**Figure 3:**
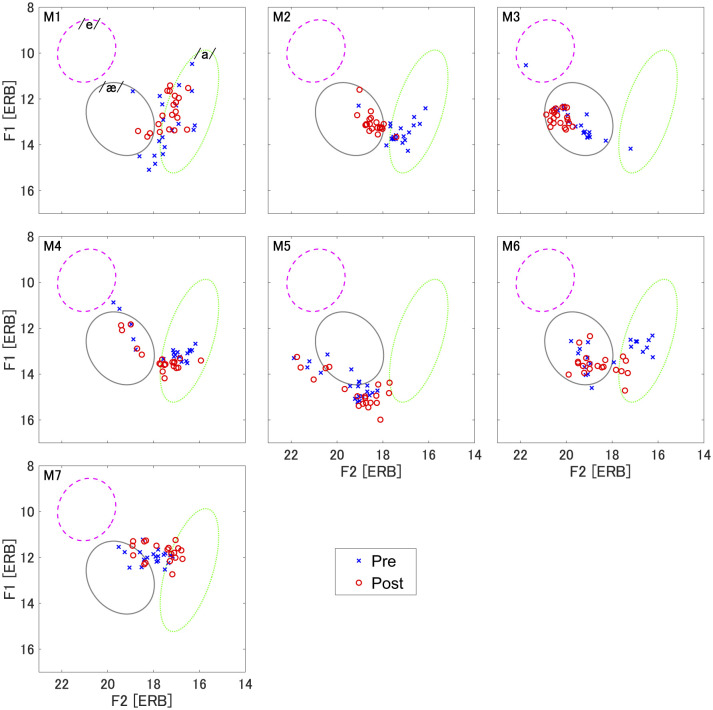
Scatterplots of utterances in the F1 × F2 space for the production of /æ/. Each panel corresponds to an individual participant (M1–M7). Blue cross marks represent the pre-training production and red circle marks are post-training production. Each mark represents individual trials. The gray ellipse represents the area considered as a typical range for the production of /æ/, obtained from the data of native speakers of AE which was used in the prediction model. The green ellipse represents the area for /a/ and the purple ellipse represents the area for /e/, which are considered as a typical range of Japanese vowels and are obtained from the data of the current seven participants recorded for the target estimation using the prediction model.

The experimental protocol for the production training session was structured into four phases, designed to systematically guide participants through the process of articulatory training and data collection:

1. **Preparation phase:** The initial phase involved the precise setup and calibration of the experimental apparatus. Each participant, fitted with sensors as depicted in Figure 1B, was seated comfortably in front of the computer screen in a quiet room. For real-time visualization, palatal shape was measured using a sensor placed on a Q-tip drawn along the midsagittal palate vault. The occlusal plane was established using sensors placed on a biteplane grasped by the speaker's teeth. Then, the participants' production of sustained Japanese vowels /a/, /i/, /ɯ/, /e/, and /o/ were recorded with five repetitions each. /a/, /i/, and /ɯ/ were used for the target estimation. /e/ was used for the visual verification of the training effect (see Figure 3). These recordings served as a basis for the subsequent estimation of each speaker's individualized articulatory target position for the AE vowel /æ/ based on the regression model.
2. **Pre-training phase:** This phase established a baseline measurement of the participants' pre-training production of the target stimuli. Participants were instructed to produce target words containing the /æ/ vowel (‘back', ‘sad', ‘had') as well as the isolated vowel /æ/ itself. These three words were chosen to be familiar to Japanese participants while providing variation in /CæC/ combinations. Each stimulus was recorded five times in randomized order, with visual instruction but without any accompanying auditory cues.
3. **Training phase:** This phase constituted the core of the articulatory training intervention. The estimated target positions for /æ/ (sensors and connecting tongue contour spline) were presented on a computer screen and participants instructed to adjust their articulators (tongue, jaw and lips) to match the displayed positions. This initial training was conducted without the production of any speech sounds (i.e., silently), for five minutes. Subsequently, they practiced producing the /æ/ vowel, prompted by the same visual feedback display of EMA sensor positions they saw in the first, silent articulation part of the training. This task, combining the newly learned articulatory movements with phonation, was repeated 20 times to reinforce the learned motor patterns and promote accurate production. This second part of the training took 5–10 minutes. Note that the participants did not hear any sample sounds produced by AE speakers during the training.
4. **Post-training phase:** The final phase mirrored the procedures of the pre-training phase. Participants again produced the target words and the isolated /æ/ vowel, following the same randomized presentation and recording protocols. This post-training data collection served to evaluate the effectiveness of the training intervention by quantifying any changes in articulatory and acoustic production relative to the pre-training baseline. Note that the estimated target positions used during the training phase were not displayed in this phase.

#### 2.3.2 Perception session

The perceptual task employed a perceptual discrimination task using an ABX paradigm. In this paradigm, participants are presented with three stimuli: A, B, and X. Stimulus X is identical to either A or B, and the participant's task is to identify which of the two it matches. The stimuli used in the perception test consisted of 75 minimal pairs contrasting the AE vowels /æ/–/ɑ/, /æ/–/ʌ/, and /ɑ/–/ʌ/ within consonant-vowel-consonant (CVC) word structures (for example, cat-cot, cat-cut, and cot-cut, see Supplementary Table 1 for all pairs). These stimuli were selected based on the following criteria: 1) maximizing phonetic variation; 2) high frequency of occurrence; and 3) minimizing the number of proper nouns included. Perception of the selected words are typically challenging for non-native English speakers, particularly those whose L1 lacks these distinctions. All stimuli were recorded by a single male native speaker of AE to ensure consistency in voice and intonation, eliminating speaker-specific variability as a potential confounding factor. Each individual stimulus was presented four times in a counterbalanced order. This counterbalancing was implemented to mitigate any potential effects of presentation order on participant responses, such as fatigue or learning effects.

In each trial of the perception test, participants listened to the three stimuli (A, B, and X) presented through headphones (Sennheiser HDA 200). Following the presentation of the three stimuli, participants were prompted to identify whether the third stimulus (X) matched the first (A) or the second (B). Responses were collected using button press on a computer keyboard. We tested all four combinations in each minimal pair. In total, 300 ABX stimuli were tested. The experimental time for the perception test was around one hour. The same procedure described in this section was applied in both perception sessions (PS-1 and PS-2).

### 2.4 Data analysis

We first tested whether the participants' production was improved by the articulatory-based training. This was followed by an examination of whether their perception acuity also changed, and whether these perceptual changes were related to the production changes induced by the training. All data analysis was carried out using MATLAB (MathWorks, Inc.) and statistical analysis was conducted using R (R Core Team, 2025).

- **Production Data:** We conducted acoustic and articulatory data analyses by comparing the pre- and post-training phases. For the acoustic analysis, F1 and F2 were extracted from the recorded audio data obtained during both the pre- and post-training phases. These formant estimates were derived using a 14th-order Linear Predictive Coding (LPC) analysis. To enhance the accuracy and stability of the formant estimates, a pre-emphasis factor of 0.97 was applied to the speech signal prior to LPC analysis. A Hanning window with a length of 25 ms and an overlap of 15 ms between consecutive frames was utilized during the LPC analysis. To ensure the reliability of the formant measurements and to avoid analyzing unstable portions of the signal, spectrograms corresponding to each utterance were visually inspected (Ladefoged, 2003). Five frames, selected from the approximate center of each utterance where spectral stability was confirmed, were averaged to obtain a representative measure for each vowel token. Given the non-linear relationship between acoustic frequency and human auditory perception, as well as the anisotropy of the F1 × F2 plane, all formant values were converted from Hz to Equivalent Rectangular Bandwidth (ERB) units (Glasberg and Moore, 1990). To assess the effectiveness of the training on production accuracy, we first carried out a visual inspection to observe differences in F1 and F2 between the pre- and post-training phases. Subsequently, to quantitatively evaluate changes in the difference from native production between the pre- and post-training phases, Mahalanobis distances were calculated between the participants' produced /æ/ formants and the distribution of /æ/ formants from native speakers, taking into account heterogeneity of formant variability in the F1 × F2 space. (Note that when F1 and F2 were normalized and found to be uncorrelated (showing uniformity in the F1 × F2 space), the Mahalanobis distance is equivalent to the Euclidean distance.) A one-way repeated measure Analysis of Variance (ANOVA) with Phase (pre- vs. post-training) as the factor was performed on a linear mixed effects model with participant as a random intercept fitted to test differences in pronunciation performance, as measured by Mahalanobis distance, between pre- and post- training phases.

For the articulatory analysis, the articulatory positions for each /æ/ utterance were characterized by averaging the recorded data points over the same time window used for the acoustic analysis. To investigate training-induced changes in articulatory positions, the distributions of articulatory positions for the pre- and post-training phases (defined by the 95% confidence limits) were computed for each measured articulatory point. To verify whether tongue position was altered between the pre- and post-training phases, Euclidean distances between the mean pre- and post-training positions were calculated for the tongue sensors (T1, T2, and T3) in the xy-plane. To test the effect of the training factor, one-sample *t*-tests were applied for each sensor to examine whether the obtained distances were significantly different from zero, thereby identifying whether a significant positional shift occurred between the two phases. As no universally effective metric has been established to represent articulatory improvements (such as distances from estimated targets) given the substantial individual variability in articulatory space, this study focuses on reporting the observed changes between the pre- and post-training phases. This approach ensures a transparent representation of the data without the introduction of potentially biased metrics.

- **Perception Data:** This analysis served as the primary means of directly addressing the current research question: whether articulatory-based training had a measurable effect on perceptual discrimination acuity. Correct answer rates were calculated as the rates that the participants correctly identified the test stimulus X in an ABX procedure. These were calculated separately for the vowel pairs including /æ/ and for the vowel pair not including /æ/. A paired *t*-test was applied to statistically assess the significance of any observed changes in discrimination performance. This was conducted separately for each vowel pair as a planned *a priori* comparison. The difference between vowel pairs is outside the scope of the current study and was not considered. Although d-prime measures were also calculated from the error rates using the loglinear correction for extreme values (Hautus, 1995), they did not materially change the distribution of results or the significance of comparisons.

## 3 Results

### 3.1 Production performance

We first show the results of acoustic analysis in the production training. Figure 3 represents data points of each individual trial in F1 × F2 space for the production of /æ/. As general tendency, most of participants showed that the sounds produced in the pre-training phase (blue cross marks in Figure 3) were biased more or less toward the Japanese vowel /a/. In four participants (M1, M2, M4, and M6), the produced sounds were mostly within range of the Japanese vowel /a/, with differences from the target AE vowel /æ/ found mainly in F2, suggesting that F2 correction is required. However, two participants (M5 and M7) produced formants slightly outside of the range of the Japanese vowel /a/. The difference from the target AE vowel /æ/ was mostly in F1, suggesting that both F1 and F2 targets needed improvement. One participant (M3) already produced the sounds within the range of the AE vowel /æ/. Participants M2 and M3 showed relatively clear production differences between the pre- and post-training phases with less overlap, whereas the rest of the participants showed partially overlapping productions between the two phases.

**Figure 4:**
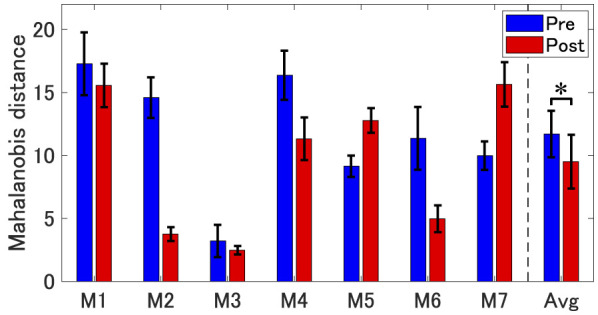
Mahalanobis distance between the participants' productions and native speakers' /æ/ sounds in the F1 × F2 space. Each bar shows the mean distance for the pre- and post-training phases, separately for individual participants (M1–M7) and for the averaged data across all participants (Avg). Error bars represent standard errors across trials (for individual participants) and across participants (for the averaged data). For Avg, * represents *p* < 0.05.

To precisely verify the difference between pre- and post-training phases, we compared the Mahalanobis distance from native speakers' utterances between the pre- and post-training phases. As shown in Figure 4, five of the seven participants (M1, M2, M3, M4, and M6) showed a decrease in acoustic distance at the post-training phase compared to the pre-training phase, while the remaining two participants (M5 and M7) showed changes in the opposite direction. These two participants corresponded to the ones who needed to improve F1. At the group level, a one-way repeated measures ANOVA based on a linear mixed effects model revealed a significant difference between pre- and post-training phases [*F*(1,271)=6.39, *p* < 0.05, partial η^2^=0.023)], suggesting improvement in pronunciation. The result demonstrated a shift closer to AE native speaker distribution in acoustic measurements (F1 and/or F2).

**Figure 5:**
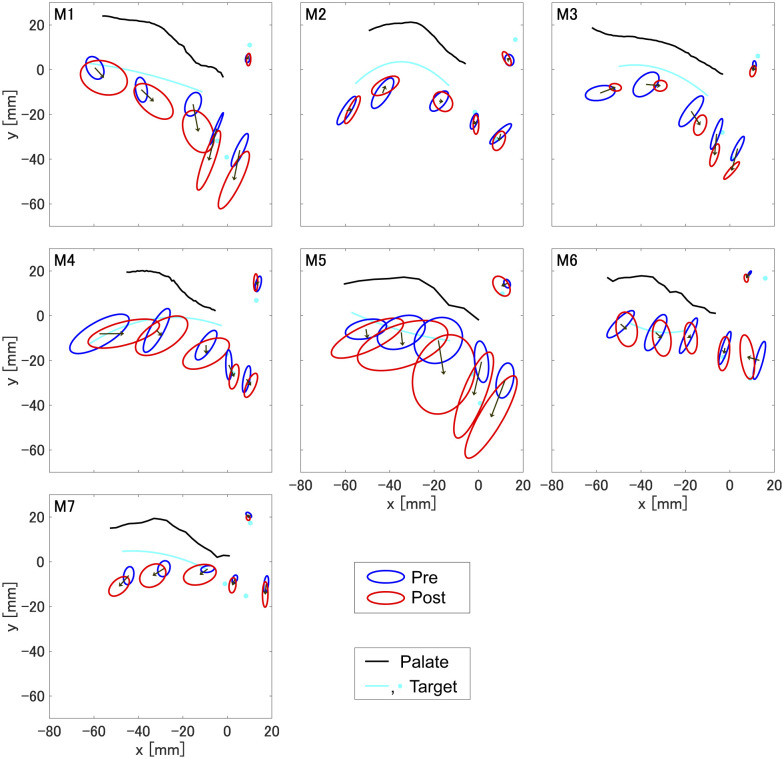
Changes in articulatory positions for the production of /æ/ between pre- and post-training phases. Each panel corresponds to each individual participant (M1–M7). The ellipses represent the 95% confidence limits of each sensor for pre-training phase (blue line) and post-training phase (red line). The black line denotes the hard palate and the cyan line and points denote the estimated target positions used during the articulatory-based training. Arrows represent the change in mean articulatory position for each sensor, connecting the centroids of the 95% confidence ellipses for pre- and post-training distributions.

Articulatory positions in pre- and post-training phases, with the estimated target used in the articulatory training, are shown in Figure 5. All participants showed changes in articulatory positions from the pre-training phase to the post-training phase. Distances between the pre- and post-training phases evaluated as *t*-tests by sensor were significantly different from zero (T1: 6.6 ± 2.0, *t*=3.26, *p* < 0.05, Cohen's *d*=1.23; T2: 5.2 ± 0.6, *t*=8.07, *p* < 0.01, Cohen's *d*=3.05; T3: 5.8 ± 1.1, *t*=5.52, *p* < 0.05, Cohen's *d*=2.09; values are mean ± standard error). This indicates that the current articulatory-based training also changed articulatory positions consistently in all participants. Although those changes were consistently observed across participants, whether and to what extent improvement occurred varied, as observed in the acoustic data.

For participants M1, M4, M5, M6, and M7, the estimated targets mostly overlapped the articulatory positions in the pre-training phase; however, the estimated targets for M2 and M3 are distinct from the articulatory positions in the pre-training phase. Although training was carried out to match articulatory positions to the estimated target, the articulatory positions in the post-training phase were not identical to the estimated targets. This indicates that the acquired articulatory positions were affected by the training but not achieved by exact alignment with the estimated target.

The estimated targets could be categorized into two patterns depending on the initial error from the native sounds. One pattern can be seen in M5 and M7 who need both F1 and F2 correction. The main improvement into the target is seen mostly in the tongue dorsum (TD) sensor. Another pattern can be seen in the rest of participants who need correction mostly in F2. In this case, the main improvement is seen either in the tongue tip (TT) sensor or over the entire tongue.

The participants who showed acoustic improvements (M1, M2, M3, M4, and M6) appeared to shift toward the estimated target, and M2 and M6 also showed post-training tongue shapes closer to the estimated target. Conversely, M5 and M7 did not show acoustic improvements, and their articulatory positions in the post-training phase were not significantly shifted to their estimated targets.

### 3.2 Perception performance

**Figure 6:**
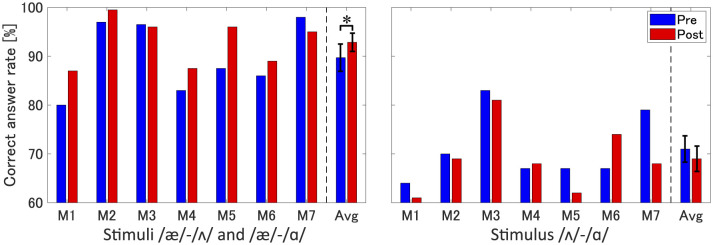
Correct answer rates for vowel perception before and after articulatory-based training in individual participants (M1–M7) and the averaged data across all participants (Avg). Left panel: vowel pairs including /æ/ (/æ/–/ɑ/ and /æ/–/ʌ/) used as stimuli. Right panel: vowel pair not including /æ/ (/ɑ/–/ʌ/). Error bars represent standard errors across participants. * represents *p* < 0.05.

Figure 6 shows the correct answer rates for the perception task: the left panel represents the vowel pairs including /æ/ (/æ/–/ɑ/ and /æ/–/ʌ/) and the right panel represents the vowel pair not including /æ/ (/ɑ/–/ʌ/). In the vowel pair including /æ/, nearly almost all participants besides M3 demonstrated improvement in the correct answer rates after the articulatory-based training. As M3 is the only participant who produces the vowel /æ/ in the acoustic range of native speakers prior to training, the perceptual change after the articulatory training can be expected to be small and limited. The participants who improved speech production due to the training (M1, M2, M4, and M6) also showed perceptual improvement. In the participants who did not improve their speech production (M5 and M7), the perceptual change varied. At the group level, statistical analysis showed a significant improvement in perceptual discrimination post-training compared to pre-training (paired *t*-test: *t*(6)=−2.07, *p* < 0.05, Cohen's *d*=0.78), suggesting the AE vowel contrasts can be more correctly distinguished after the training.

In the vowel pair (/ɑ/–/ʌ/), the perceptual change varied across all participants. The average data did not show any significant differences (paired *t*-test: *t*(6)=0.96, *p*=0.37, Cohen's *d*=0.36), suggesting that the /æ/–focused articulatory training did not affect the irrelevant vowel contrast. The results support the hypothesis that production training enhances perceptual performance, particularly for sounds targeted in training.

## 4 Discussion

In our previous study (Suemitsu et al., 2015), we developed a real-time visual feedback approach using EMA for articulatory target-based (ATB) L2 training. We applied this system to pronunciation learning of the AE vowel /æ/ for Japanese learners of AE and found the training shifted their production into more native-like patterns. Given expected perception-production links, in the current study we hypothesized that the ATB training for speech production would also modify perceptual acuity for the trained vowel. To verify this hypothesis, we carried out a perceptual task before and after ATB training. To eliminate possible influence from an acoustical learning process, the current training involves a solely articulatory presentation without model target sounds given during the training. As found in the previous study, ATB training improved production ability for the AE vowel /æ/. By comparing perception acuity between pre- and post-training phases, improvement was found only in the vowel pair including /æ/ and mostly in the participants who also improved their production. Since no acoustical cues were involved in the articulatory training sessions, this perceptual improvement derives from articulatory-based modulation.

The results suggest that improved articulatory control likely provides L2 learners with a more stable representation of L2 sounds, facilitating better perceptual mapping. The findings align with theoretical view that speech motor learning enhances auditory discrimination.

### 4.1 Perceptual improvement due to articulatory-based training

Speech production and perception are closely linked and likely interact during language acquisition. The current results support this idea by showing that changes in speech production affected speech perception in L2. Although one participant (M3) showed little or no change in speech perception, this may be because his productions were already within the range of /æ/ produced by AE speakers. This case may occur, but it appears to be exceptional, possibly reflecting either prior exposure to AE or a superior ability to reproduce novel L2 speech sounds after brief exposure to them. The remaining participants showed pre-training productions outside of or barely within AE speaker targets, and all showed changes in their speech production post-training and corresponding modulation of speech perception. When speech production improved, speech perception also improved. M5 did not show a significant shift in production, but his tongue position changed in the direction of native production, and his perception improved. Conversely, M7 shifted away from native production, and his perception actually deteriorated. These results suggest that the speech production training can play an important role in the enhancement of L2 speech perception.

Effects of production training on speech perception have been frequently investigated in the context of *implicit* training based on altered auditory feedback (Shiller et al., 2009; Lametti et al., 2010; Ohashi and Ito, 2019). Auditory errors are implicitly imposed by manipulating the produced sounds with real-time alteration of formants in such training paradigm. Participants adapt to the implicitly imposed environmental change by modifying their production, and this adaptive change in speech production can also result in the modulation of their speech perception. Here, however, we applied an *explicit* training procedure in which participants were asked to adjust their articulatory positions using an explicitly imposed target. In this explicit training, improvement of speech production through training also resulted in improvement in speech perception, consistent with previous studies (Li, 2024; Stratton, 2025). These results suggest that, like implicit motor learning, explicit motor learning also plays an important role in improving speech perception in L2 acquisition.

### 4.2 Implications for L2 acquisition

Explicit learning is conventionally used in speech learning contexts, including L2 learning. The typical method is to provide articulatory information, verbally or by showing drawings of tongue posture examples for vowel production. For this to be useful, L2 learners need to also learn how to translate this information onto articulatory movement, and learning this mapping can take a long time. As shown in the previous study (Stratton, 2025), while this type of explicit learning did improve both speech production and perception, six 20-minutes sessions were carried out during 3 to 6 weeks. Li (2024) also shows the improvement of production and perception performance using observation-based production training. This training applied to six 45-minutes sessions. In contrast, the ATB training described here used a single-session direct presentation of an articulatory target, with feedback presented in real-time, and achieved similar improvements in production and perception. Accordingly, learning driven in this way could potentially be achieved with relatively short training periods. In the current case, less than 15 minutes of the training improved both L2 vowel production and perception.

In addition, acoustical presentation of model sounds is the most commonly used training method, presented solely or in combination with articulatory information. Here, however, training is articulatory alone without acoustical presentation. This is consistent with a previous study (Li, 2024) showing that observation-based production training of articulation with minimal involvement of the acoustical model improved both production and perception more than perception based training alone. In that approach, however, production training is a conventional imitation procedure in which the learner tried to imitate the instructor's articulatory movement. Our current result suggests that ATB training can be effective for L2 learning in both speech production and perception despite its short training period.

### 4.3 L1 interference

L1 interference is a significant issue for L2 acquisition. The production of L2 vowels can be affected by the L1 vowel system when that vowel is not present in L1 (Best, 1995; Best and Tyler, 2007). In the current case of Japanese learners of AE, the American vowel /æ/ was initially produced mostly as a sound near the Japanese vowel /a/, as seen in Figure 3 (especially M1, M2, M4, and M6). To the extent that these participants are representative, F2 can be taken as the main correction parameter for Japanese learners of AE, and the difference in F2 was improved by the articulatory-based training. But we also observed a different outcome, in which the initial production of /æ/ was shifted along F1, and both F1 and F2 needed adjustment (M5 and M7). In contrast to the typical cases, these two participants did not improve toward the target vowel acoustically. These differing training effects may be related to limitations of our current training method. In the case requiring only F2 correction, the target tongue posture was estimated so as to make a smaller constriction between the tongue tip and palate than the speaker's initial position. For this adjustment, precise control of the tongue tip is required. In contrast, for corrections including F1, the target estimate also modified the tongue dorsum. Coordinating both tongue dorsum and tip targets is more difficult than modifying the tongue tip alone. This was confirmed by our participants who reported that it was easier to adjust the tongue tip to the estimated target than the tongue dorsum.

It is challenging to estimate the articulatory target of a novel vowel for L2 learners. The current estimation model was based on our empirical assumption of one type of L1 interference, namely, that a gap in L1 inventory (in this case /æ/) could be filled given training with an appropriate articulatory target. However, a different form of interference occurs when no gaps exist, and the L2 target overlaps with a similar but not identical L1 target (e.g., Japanese /e/ vs. AE /ε/). We need to further improve our estimation methods to generalize our training approach to interference of this type.

### 4.4 Limitations

It is possible that the tongue dorsum may have been inadequately estimated due to the limited number and anterior placement of the EMA sensors (placement posterior to the TD sensor position is not feasible due to gag responses). Moreover, our approach to estimating target positions may also have limitations. In this method, the vowels /a/, /i/, and /ɯ/ were used for references mapping between Japanese and AE. However, these vowels, although similar, are not identical acoustically nor articulatorily, and consequently the estimate for /æ/ may be affected accordingly. Moreover, both the source data and the feedback data are midsagittal, and do not take possible effects of parasagittal vocal tract morphology into account. In future work, inclusion of an additional estimation vowel (/e/, which exists in both Japanese and AE) may mitigate some of these concerns.

In this study, the relatively small sample size may limit the reliability of the findings. Nevertheless, all participants exhibited measurable changes in their articulatory positions after a short period of articulatory training and speech perception was also modified following this training. The observation of individual participants further indicated that improvements in speech production resulted in corresponding improvement in speech perception. These results strongly suggest that explicit targeted motor training can improve both speech perception and production.

For further confirmation of the robustness of the observed effects, testing a larger and more diverse group of participants is necessary. It would also be valuable to conduct longitudinal follow-up experiments to assess the long-term retention of both production and perception improvements. The current training focused solely on a single vowel (/æ/) as a representative target. Expanding into other vowels or consonants would help determine whether the observed effects can be generalized to a broader range of L2 phonetic categories.

## 5 Conclusion

This study demonstrates that articulatory-based pronunciation training improves both production and perception of L2 sounds. Enhanced articulatory control of the AE vowel /æ/ translated into better perceptual discrimination of related contrasts. These findings highlight the interconnected nature of speech production and perception, and support the integration of motor-based feedback in language learning methodologies for L2 pedagogy. This improvement, achieved without model sounds, suggests that motor-based training can be more effective than perception-only training. Further exploration is warranted to optimize training designs and evaluate their application across diverse linguistic contexts.

## Data Availability

Anonymized data supporting this work will be provided upon written request to the lead author.
