# Supplementary material for: Improvement of speech perception following articulatory-target based production training for second language acquisition
Source: Front Hum Neurosci. 2026 Apr 13;20:1743034. doi: 10.3389/fnhum.2026.1743034 (PMC13111571; doi:10.3389/fnhum.2026.1743034)
Supplement: Supplementary file 1 [file Data_Sheet_1.docx]

Supplementary Material

# Supplementary Figures and Tables

## Supplementary Figures


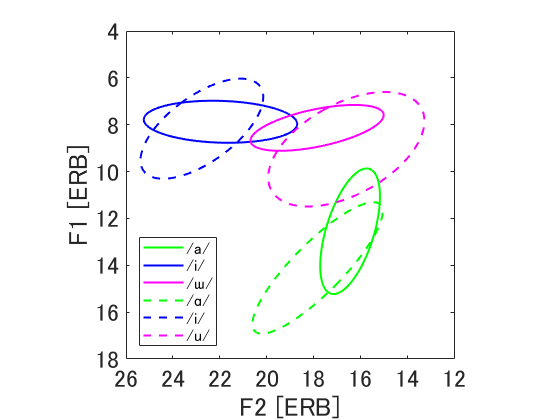


**Supplementary Figure 1.** Comparison of vowel distributions between /a/, /i/, and /ɯ/ in Japanese and /ɑ/, /i/, and /u/ in AE. Ellipses represent the 95% confidence intervals for each vowel. Solid contour denotes AE vowels obtained from our dataset of AE speakers used for the prediction model (24 males and 25 females) and dashed contour denotes Japanese vowels obtained from the current participants (7 males).

## Supplementary Tables

**Supplementary Table 1.** All stimuli used in the perception test.

|  | /æ/-/ɑ/ condition | /æ/-/ʌ/ condition | /ɑ/-/ʌ/ condition |
| --- | --- | --- | --- |
| 1 | cab – cob | cab – cub | cob – cub |
| 2 | nab – knob | nab – nub | knob – nub |
| 3 | cad – cod | cad – cud | cod – cud |
| 4 | bad – bod | bad – bud | bod – bud |
| 5 | mad – mod | mad – mud | mod – mud |
| 6 | sad – sod | sad – sudd | sod – sudd |
| 7 | pap – pop | pap – pup | pop – pup |
| 8 | cap – cop | cap – cup | cop – cup |
| 9 | sap – sop | sap – sup | sop – sup |
| 10 | gat – got | gat – gut | got – gut |
| 11 | pat – pot | pat – putt | pot – putt |
| 12 | cat – cot | cat – cut | cot – cut |
| 13 | gnat – knot | gnat – nut | knot – nut |
| 14 | rat – rot | rat – rut | rot – rut |
| 15 | hat – hot | hat – hut | hot – hut |
| 16 | pack – pock | pack – puck | pock – puck |
| 17 | tack – tock | tack – tuck | tock – tuck |
| 18 | Mac – mock | Mac – muck | mock – muck |
| 19 | lack – lock | lack – luck | lock – luck |
| 20 | rack – rock | rack – ruck | rock – ruck |
| 21 | sack – sock | sack – suck | sock – suck |
| 22 | shack – shock | shack – shuck | shock – shuck |
| 23 | hack – hock | hack – Huck | hock – Huck |
| 24 | dam – Dom | dam – dumb | Dom – dumb |
| 25 | mam – Mom | mam – mum | Mom – mum |
